# Iron Deficiency among School-Aged Adolescents in Hong Kong: Prevalence, Predictors, and Effects on Health-Related Quality of Life

**DOI:** 10.3390/ijerph20032578

**Published:** 2023-01-31

**Authors:** Yin Ting Cheung, Dorothy Fung Ying Chan, Cheuk Kwong Lee, Wai Chiu Tsoi, Ching Wa Lau, Jennifer Ngar Sze Leung, Jason Chi Chiu So, Chris Lei Po Wong, Stella Tsui Ying Tsang, Yvonne Yuen Ling Chu, Chi Kong Li

**Affiliations:** 1 School of Pharmacy, The Chinese University of Hong Kong, Hong Kong SAR, China; 2 Department of Paediatrics, Prince of Wales Hospital, Hong Kong SAR, China; 3 Hong Kong Red Cross Blood Transfusion Service, Hong Kong SAR, China; 4 Department of Pathology, Hong Kong Children’s Hospital, Hong Kong SAR, China; 5 Hong Kong Molecular Pathology Diagnostic Centre, Hong Kong SAR, China; 6 Department of Paediatrics, The Chinese University of Hong Kong, Hong Kong SAR, China; 7 Department of Paediatrics and Adolescent Medicine, Hong Kong Children’s Hospital, Hong Kong SAR, China; 8 Hong Kong Hub of Paediatric Excellence, The Chinese University of Hong Kong, Hong Kong SAR, China

**Keywords:** iron deficiency, anemia, adolescents, dietary, quality of life

## Abstract

Iron deficiency (ID) is a prevalent nutritional deficiency affecting children/adolescents worldwide. We reported (1) the prevalence of ID and ID with anemia (IDA) among Chinese school-aged adolescents, (2) clinical and dietary predictors of iron status, and (3) its impact on health-related qualities of life (HRQoL). This cross-sectional study recruited 183 boys and 340 girls (mean age = 17.55) from 16 schools in Hong Kong. ID is defined as serum ferritin <15 μg/L. The participants reported their dietary habits, menstrual patterns (girls), and HRQoL using structured questionnaires. The overall prevalence of ID was 11.1%. None of the boys had ID or IDA. Among girls, the rate of ID was 17.1% and IDA was 10.9%. One-third (36.3%) reported a regular habit of skipping ≥1 meal/day. Lower ferritin was found in adolescents who skipped meals (Est = −35.1, *p* = 0.017). Lower ferritin is correlated with poorer school functioning (Est = 0.81, *p* = 0.045) and fatigue (Est = 0.92, *p* = 0.016). Skipping meals is associated with poorer physical (*p* = 0.0017) and school functioning (*p* = 0.027). To conclude, 1 in 10 school-aged adolescents in Hong Kong are iron-deficient. The ID rate in girls (17.1%) is similar to that in other industrialized countries (5.2–16.6%). Future work should promote awareness on the potential health consequences of poor dietary habits on ID and the well-being of adolescents.

## 1. Introduction

Iron deficiency (ID) is one of the most prevalent nutritional deficiencies affecting children and adolescents worldwide. According to the latest Global Burden of Disease study of children and adolescents, iron-deficiency anemia is the only nonfatal disease ranked within the top 10 global leading causes of disability-adjusted life years [1]. The age-standardized rate of ID is 25.1% globally and, strikingly, there is not much difference between developed and developing countries [2].

The reported age-standardized rate of ID in China was 22.6% between 1990 and 2013 [2]. In a recent study that covered 26 provinces and four municipalities, ID was observed in 10% of the girls and 7% of the boys in the study cohort [3]. Adolescents are at a particular risk of ID due to the increased iron intake required for pubertal growth, especially in girls [4,5]. The gastrointestinal tract plays a major role in iron absorption, which is dependent on the form and amount of iron in the diet [6]. Adolescents are at high risk for developing ID as the accelerated increase in iron requirements during puberty and rapid growth might be unbalanced by inadequate dietary iron intake and chronic blood loss (menstruation in girls) [7].

ID in the presence of a low hemoglobin level is known as ID anemia (IDA). Iron is an essential micronutrient in DNA synthesis, energy production, and erythropoiesis. Other than playing an indispensable role in hemoglobin synthesis, iron is also needed for the synthesis of proteins/enzymes involved in vital biological processes through the production of heme and iron–sulfur clusters [8]. Iron also plays a critical role in neurotransmitter synthesis and neurodevelopment [9]. Although anemia is the most evident sign of ID, the impact of ID in organs/tissues other than those involved in erythropoiesis often precedes the onset of anemia [10]. Studies have reported that ID, even without anemia, might manifest as fatigue, poor concentration, sleep disorders, and neurological symptoms such as restless leg syndrome [11,12,13,14]. These health consequences of ID can lead to functional limitations in the daily lives of affected individuals.

In 2012, a study of around 200 young adult blood donors (from 21 to 35 years old) in Hong Kong found low serum ferritin levels (<10 μg/L) in 7.2% of the female donors [15]. However, to date, no local study has evaluated the prevalence of ID and IDA and its effect on functional outcomes, such as health-related quality of life (HRQoL) and fatigue, among school-aged adolescents. ID might potentially affect many aspects of an adolescent’s HRQoL, such as his/her physical, emotional, cognitive, and school functioning [16]. Such a study is necessary to evaluate whether the national initiatives of food fortification and complementary public health measures of the past decade have succeeded in preventing and controlling ID and IDA among adolescents.

The objectives of this study were to (1) determine the prevalence of ID and IDA among school-aged adolescents in Hong Kong, (2) identify the dietary predictors of iron status, and (3) evaluate the association between iron status and functional outcomes (HRQoL and fatigue) in this population. We hypothesized that (1) adolescents with lower dietary iron and heavy menstrual bleeding (for girls) would have lower iron stores, as reflected by a lower serum ferritin level and (2) lower iron stores were associated with poorer self-reported HRQoL and more fatigue.

## 2. Methods

This was a cross-sectional study conducted at public secondary schools in the community. Between October 2020 and December 2021, normal healthy adolescents were recruited through blood donation campaigns run by the Red Cross Blood Transfusion Service. This study was approved by the Joint Chinese University of Hong Kong–New Territories East Cluster Clinical Research Ethics Committee. A study schema is presented in Supplementary Figure S1.

### 2.1. Study Population

Eligible adolescents were recruited if they were between 16 and 19 years of age and had undergone blood donation screening. The adolescents who were not fit to donate blood following the screening were still eligible to participate in this study. Eligible adolescents had to understand written Chinese, which is the medium of instruction for the vast majority of the public schools in Hong Kong.

We excluded adolescents who showed signs or symptoms of an active infection, reported a history of anemia, or were currently receiving treatment for anemia. The participants who were 18 years old or over provided written informed consent, while those who were under 18 provided written assent and informed consent from their parents.

### 2.2. Definitions of ID and IDA

Upon recruitment, approximately 10 mL of blood was collected from each participant under fed conditions, as they were advised to have adequate food and fluid intake before undergoing blood donation. The samples were sent to the Department of Pathology Laboratory at the Hong Kong Children’s Hospital on the same day. The serum ferritin level and complete blood count were measured. The C-reactive protein (CRP) levels were also measured to exclude inflammatory conditions that lead to elevated serum ferritin and any samples with CRP > 9 mg/dL were excluded. The specifications of the instruments and tests are presented in Supplementary Table S1.

A serum ferritin level of <15 μg/L is considered ID in both boys and girls (World Health Organization [WHO] definition) [17]. IDA is defined as the presence of both ID and anemia. In this study, two different definitions of anemia were used. First, as per the WHO, anemia was defined as a hemoglobin level of <12 g/dL in girls and a hemoglobin level of <13 g/dL in boys [18]. We also conducted a sensitivity analysis using a lower threshold, as thalassemia is a common condition associated with low hemoglobin levels in the Asian population [19]. This lower threshold (the local definition of anemia) was a hemoglobin level of <11.5 g/dL in girls and <12 g/dL in boys.

Additional molecular testing for the common thalassemia mutation was performed in adolescents who presented with low mean red cell volume and anemia. Consistent with a local landmark study [19], the WHO’s definition of anemia was adopted for thalassemia genotyping to detect causes of anemia among individuals who had borderline low hemoglobin levels.

For ethical purposes, the adolescents who were diagnosed with IDA, or their parents/legal guardians, were contacted and offered additional consultation with a hematologist.

### 2.3. Factors Associated with Iron Stores

The participants completed a structured questionnaire to report their dietary patterns. They reported whether they tended to skip meals (breakfast, lunch, or dinner) and the frequency with which they consumed common iron-rich foods (Supplementary Table S2). A dietary iron score was then computed. This score was a composite of the type and frequency of food intake and drew on information provided by the Hong Kong Department of Health about the typical iron content per serving of various foods [20]. The computed scores ranged from 36 to 144 points, with a higher score indicative of higher dietary iron from food sources. More details on the tabulation of the dietary iron score can be found in Supplementary Table S2.

Among female adolescents, menstrual characteristics were self-reported using a structured questionnaire. To gather increased detail on heavy flow, the participants were asked about the number of sanitary products they used on the heaviest day of their cycle and the average bleeding severity.

The participants’ weights were measured using a standing digital medical-grade weighing scale as part of the blood donation screening procedure. The weights were measured to the nearest 0.1 kg. They self-reported their height in centimeters. Body mass index (BMI, kg/m^2^) was calculated as the weight in kilograms divided by the height in meters squared. BMI percentiles were determined using growth charts from the Centers for Disease Control and Prevention [21]. The participants were classified according to age- and sex-adjusted BMI percentiles. Normal weight was defined as a BMI between >10th and <85th percentiles, underweight as ≤10th percentile, overweight as ≥85th and <95th percentiles, and obese as ≥95th percentile.

### 2.4. Functional Outcomes

The Pediatric Quality of Life (PedsQL) 4.0 was used to measure the adolescents’ HRQoL. The 23-item PedsQL 4.0 Generic Core Scales consist of separate scales measuring physical functioning, emotional functioning, social functioning, and school functioning. The PedsQL has been validated in Chinese adolescents [22].

Fatigue was measured using the PedsQL Multidimensional Fatigue Scale (MDS) for adolescents aged from 13 to 18 years old. The subscales of this instrument are general fatigue, sleep fatigue, and cognitive fatigue. The Chinese version of the MDS has demonstrated good internal consistency reliability, content validity, and construct validity in the Chinese adolescent and young adult population [23].

### 2.5. Statistical Analysis

The hematological characteristics of the cohort were summarized using descriptive analysis. Multivariable general linear models (GLMs) were constructed to evaluate the association between the serum ferritin level and predictors: skipping meals and self-reported dietary iron intake, weight status, menstrual bleeding severity, and average number of sanitary products used on the heaviest day (for female adolescents). All the GLMs were adjusted for age and sex. The estimates and standard errors (SEs) of the GLMs are reported.

Finally, the GLMs were constructed to identify the associations with functional outcomes (HRQoL and fatigue measures), using serum ferritin and dietary factors as independent predictors and adjusting for age and sex. All the statistical tests were two-tailed and a *p*-value of <0.05 was considered statistically significant.

## 3. Results

The sample consisted of 523 adolescents who were recruited from 16 schools during the blood donation drive. Twenty-nine of these were deferred for blood donation due to low hemoglobin levels but still completed all the functional assessments. The mean age at baseline was 17.55 (SD = 1.05) years (Table 1). Two-thirds of the participants were female (*n* = 340, 65.0%).

### 3.1. Hematological Findings in the Overall Cohort

A summary of the hematological parameters of the study cohort is presented in Supplementary Table S3. The mean hemoglobin for the overall cohort was 13.25 g/dL (SD = 1.4, range 6.9–17.1 g/dL). The mean MCV was 86.2 (SD = 6.6, range 56.4–101.6). The mean serum ferritin was 94.6 μg/L (SD = 86.7, range 2.2–608.8).

In total, 58 adolescents (11.1%) had serum ferritin levels of <15 μg/L and were considered ID (Table 2). For the overall cohort, the rate of IDA is 7.1% based on the WHO definition and 4.4% based on the local definition. None of the participants showed elevated CRP levels.

### 3.2. Hematological Findings in Boys

**Figure 1:**
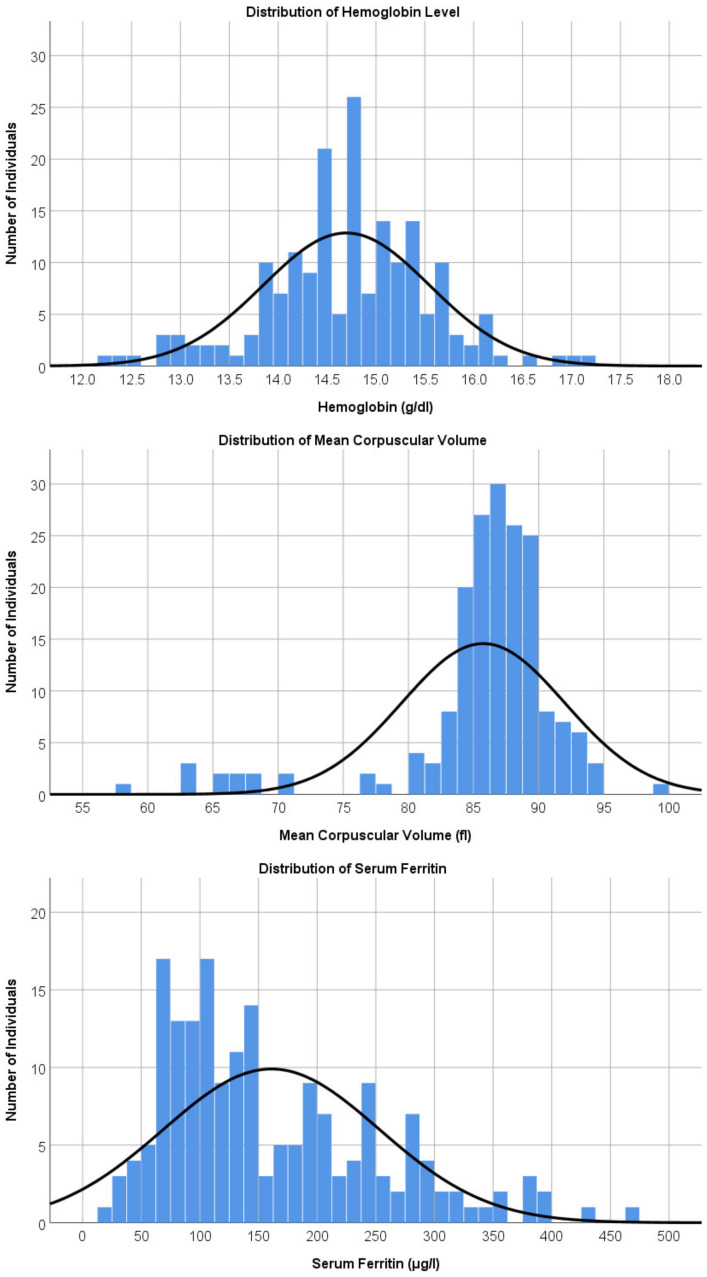
Distribution of hematological findings in male adolescents. Caption: Distribution of hemoglobin level, mean corpuscular volume, and serum ferritin levels in boys. One boy with a serum ferritin level of 608.8 μg/L was excluded from the histogram plot.

The mean hemoglobin level of the boys (*n* = 183) was 14.7 g/dL (SD = 0.85, range 12.2–17.1; Figure 1) and the mean MCV was 85.7 (SD = 6.3, range 57.5–99.8; Figure 1). The mean serum ferritin was 160.9 μg/L (SD = 92.1, range 19.1–472.2; Figure 1). None of the male participants had serum ferritin levels of <15 μg/L.

None of the boys were classified as having anemia using the local definition, as all of them had hemoglobin levels of ≥12 g/dL. However, seven of the boys (3.8%) had hemoglobin levels between 12.2 g/dL and 12.9 g/dL and were therefore defined as anemic based on the WHO’s definition. A subsequent thalassemia genotyping test conducted on six of the seven participants revealed that three of them had the alpha thalassemia trait and three had the beta thalassemia trait.

### 3.3. Hematological Findings in Girls

**Figure 2:**
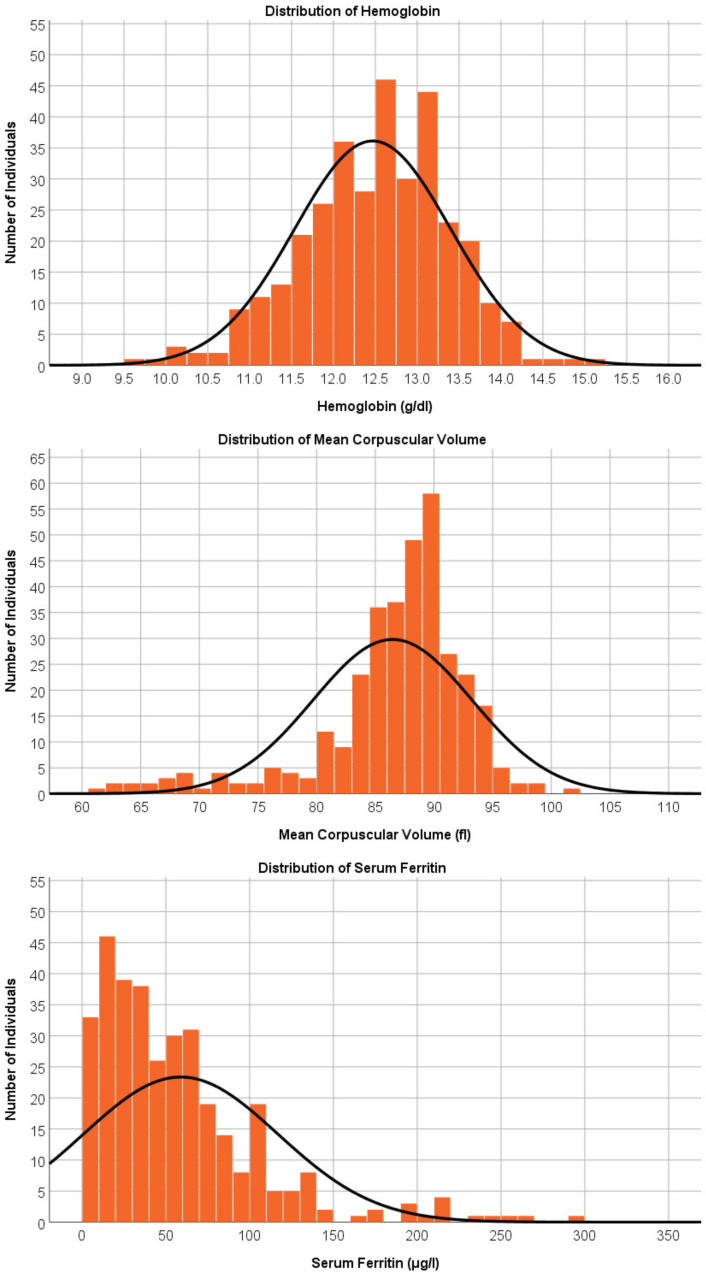
Distribution of hematological findings in female adolescents. Caption: Distribution of hemoglobin level, mean corpuscular volume, and serum ferritin levels in girls. One girl with a hemoglobin level of 6.9 g/dL was excluded from the histogram plot.

The mean hemoglobin level of the girls (*n* = 340) was 12.5 g/dL (SD = 0.93, range 6.9–15.0; Figure 2) and the mean MCV was 86.4 (SD = 6.8, range 56.4–101.6; Figure 2). A hemoglobin level of <11.5 g/dL was found in 43 (12.7%) of the girls. Of these 43 girls with anemia, 25 also presented with microcytosis (MCV < 80).

The mean serum ferritin was 58.9 μg/L (SD = 58.1, range 2.2–608.8; Figure 2). ID was found in 58 (17.1%) of the girls with serum ferritin <15 μg/L. In this group, 23 girls had hemoglobin levels of <11.5 g/dL. Therefore, 6.8% of the girls had IDA according to the local definition of anemia.

Based on the WHO definition of anemia, a hemoglobin level <12 g/dL was found in 91 (26.8%) of the girls, of whom 37 (10.9%) also had ID and, therefore, had IDA based on the WHO definition. Twenty of the girls in the IDA group underwent thalassemia trait genotyping. Only three of them had the alpha thalassemia trait, the other 17 were negative for both the alpha and the beta thalassemia traits.

### 3.4. Association between Dietary Characteristics and Iron Store

When asked about their dietary patterns, 189 (36.3%) of the participants (males: 28.4%, females: 40.6%) reported that they had a regular habit of skipping at least one meal per day. Breakfast was the most commonly missed meal (*n* = 149, 28.5%) (Table 3).

The participants who skipped at least one meal per day had lower serum ferritin levels than the participants who did not report a regular habit of skipping meals (median [IQR] 54.5 [23.1–105.0] vs. 73.9 [39.2–136.2] μg/L; *p* = 0.017, Table 3). Lower serum ferritin levels were also found in the participants who skipped breakfast (*p* = 0.019) and lunch (*p* = 0.011), but not dinner (*p* = 0.66), in the multivariable analyses (Table 3).

The average dietary iron intake score was 97.78 [16.10] points for the overall cohort. No significant association was found between the self-reported dietary intake and serum ferritin levels (est. = 0.74, SE = 0.43; *p* = 0.091) (Table 3).

### 3.5. Association between Weight Status and Iron Store

Overall, 13.5% of the participants were overweight/obese (males: 20.8%, females: 9.7%). As compared with the participants with normal weight, the participants who were overweight/obese had higher serum ferritin levels (median [IQR] 64.9 [31.1–109.9] vs. 106.8 [40.9–235.8] μg/L; *p* < 0.0001) in the multivariable analysis (Table 3). No significant difference was observed in the serum ferritin levels of the participants with normal weight vs. the participants who were underweight.

### 3.6. Association between Menstrual Characteristics and Iron Store

Of the female participants, 85.2% (*n* = 288) reported from little to moderate bleeding every cycle, while 14.8% (*n* = 50) reported heavy or very heavy bleeding (Table 3). The average number of sanitary products used on the heaviest day of the cycle was 3.98 [1.41]. The female participants who reported heavy or very heavy bleeding had lower serum ferritin levels than the female participants who reported from little to moderate bleeding (median [IQR] 34.0 [20.0–56.0] μg/L vs. 45.8 [20.9–77.4] μg/L; *p* = 0.028). After adjusting for age, the average number of sanitary products (est. = −13.7, SE = 5.1; *p* = 0.0072) and bleeding severity (est. = −40.7, SE = 20.1; *p* = 0.043) were associated with serum ferritin levels in female participants.

### 3.7. Association between Iron Store and HRQoL

The descriptive characteristics for HRQoL and fatigue in the overall cohort are presented in Supplementary Table S4. After adjusting for age and sex, lower serum ferritin levels (per 10 μg/L) were significantly correlated with poorer HRQoL in terms of physical functioning (est. = 0.78, SE = 0.27; *p* = 0.0047) and school functioning (est. = 0.81, SE = 0.40; *p* = 0.045; Table 4). No significant associations were observed for emotion, social, and overall psychosocial functioning.

The participants who had a habit of skipping at least one meal per day reported poorer physical functioning (*p* = 0.0017), emotional functioning (*p* = 0.026), and school functioning (*p* = 0.027). The participants who reported a lower dietary iron intake also reported poorer physical functioning (*p* = 0.0014) and school functioning (*p* = 0.010).

### 3.8. Association between Iron Store and Fatigue

After adjusting for age and sex, lower serum ferritin levels (per 10 μg/L) were significantly correlated with general fatigue (est. = 0.92, SE = 0.38; *p* = 0.016; Table 5). No significant associations were observed between serum ferritin and sleep or cognitive fatigue. The participants who had a habit of skipping at least one meal per day reported more sleep-related fatigue (*p* = 0.0038).

## 4. Discussion

This is the first study to capture the prevalence of ID and IDA among adolescents aged from 16 to 19 years old in Hong Kong. Due to its documented effects on physical and functional outcomes, ID and IDA in adolescents are important public health concerns. In this study, the prevalence of ID in the overall cohort was 11.1%. Among the girls, the rate was 17.1%. Under the local and WHO definitions of anemia, the rates of IDA in the girls were 6.8% and 10.9%, respectively. None of the boys were found to have ID or IDA. Unhealthy eating habits were associated with lower iron stores, which may explain their adverse effects on physical and school functioning.

The rate of IDA (7.1%) reported in our study is lower than the global estimate (25.1%) and estimates from other developed countries (21.3%) [2]. However, our results suggest that one in every ten adolescents in the community might have ID with or without anemia and this observation warrants attention from a public health perspective. Meal skipping was related to suboptimal iron intake, a finding that concurs with previous findings that meal skipping contributes to poor micronutrient intake in adolescents [24,25]. Breakfast was the most commonly skipped meal. This is worrying, as breakfast provides a unique opportunity to consume important micronutrients that may be less present in other meals of the day, particularly vitamins and minerals [26].Future studies should investigate the socio-environmental factors (e.g., residential district, family income, and single-parent families) that influence low iron stores and poor dietary habits. More generally, our results support the need for continued efforts from the government, schools, and nonprofit organizations to improve diet quality among Hong Kong children and adolescents.

Interestingly, the prevalence of ID among girls in our study (17.1%) was more than twice higher than the estimate of 7.2% reported in a local study conducted in 2012 in a similar blood donation setting [15]. Although the latter study had adopted a lower ID definition of serum ferritin <10 μg/L, our findings are still surprising, as one might expect nutrient standards to have improved over the last decade. The rate of ID among the girls in our study is similar to the higher end of the prevalence rates in other industrialized countries, which range from 5.2% to 16.6% [27]. This might be attributed to unhealthy eating habits, as we found that 40% of the girls reported a habit of skipping at least one meal per day. Studies have reported that female adolescents have poorer eating habits than male adolescents because they tend to be preoccupied with their physical appearances [28,29]. This phenomenon is even more prominent in Asian settings, in which societal norms may lead to an even more distorted perception of weight and body image satisfaction than in Western cultures [29,30]. Consistent with the literature [14,31], we also observed that girls who reported heavy menstrual bleeding had lower iron stores. Together, all these findings suggest that it would be beneficial to promote public health initiatives that emphasize healthy dietary habits among adolescent girls and promote awareness of the health consequences of menstrual disorders. This will encourage affected girls to seek medical attention before the anemic symptoms manifest as functional impairments.

Some studies in the literature have reported that obese or overweight adolescents are at risk of developing ID, possibly due to the upregulation of hepcidin synthesis by leptin and diminished iron stores from the accumulation of subcutaneous adipose tissue [32,33]. However, our study observed that adolescents who were obese or overweight had higher serum ferritin levels than those with normal weights. This finding should be interpreted cautiously due to selection bias, as prospective blood donors tended to be more health-conscious and have a better health state. Height was self-reported by the adolescents and not objectively measured, hence there might be potential bias in their reporting and inaccuracy in the calculation of BMI. To note, 12% of the cohort were underweight. Although we did not observe significant differences in serum ferritin levels between underweight and normal weight individuals, future studies should include a more in-depth investigation of the dietary patterns and the socioeconomic risk factors of poor nutrition intake in adolescents who require weight management interventions.

We attempted to explore the impact of low iron reserve on downstream functional outcomes in adolescents. For this analysis, we used iron serum ferritin as the predictor of interest, instead of comparing HRQoL and fatigue outcomes between individuals with ID or IDA vs. individuals without ID or IDA. This is because the clinical thresholds used in the diagnosis of ID or IDA might not apply when we evaluate the impact of iron on functional outcomes. Even if an adolescent is not clinically diagnosed with ID or IDA, a low–normal ferritin level might still affect his/her functional outcomes as studies have shown that the impact of ID in other organs/tissues (other than those involved in erythropoiesis) and neurodevelopment may occur even before the ID manifests as anemia [10]. In our study, lower ferritin levels were associated with poorer physical functioning and fatigue, a finding that concurs with findings from the literature [14,34]. Poorer school functioning might affect the downstream learning and academic performance of students. For example, one study conducted with 5398 school-aged children and adolescents in the United States showed lower standardized mathematics scores in iron-deficient school-aged children and adolescents compared to those with a normal iron status [35]. Another study found poorer academic achievement among young women with ID [36]. The adverse impact of low iron stores on school functioning is especially relevant in Hong Kong, where the local education system is highly examination oriented. The adolescents in our study were probably experiencing high levels of academic stress while preparing for the national Hong Kong Diploma of Secondary Education Examination [37]. As ID is highly correctable with oral iron supplements and an iron-fortified diet, the identification of individuals with ID is an important educational issue as well as a public health issue. Future research should aim to evaluate more precisely the impact of ID and IDA on cognitive performance and academic achievement in local adolescents.

Despite its relatively large cohort of school-aged adolescents and well-characterized hematological assessment, this study had several important limitations. First, we recruited adolescents who were participating in the blood donation program at the local secondary schools. This might have led to selection bias, as blood donors have been found to have increased altruism, higher socioeconomic status, and better access to health communication than non-donors [38] Given that non-donors’ perceptions of their health might be worse than donors’ perceptions, the actual prevalence of ID and IDA might be even higher than our estimates. Second, social desirability and recall bias may have affected the accuracy of the self-reported results. We did not use a comprehensive questionnaire to assess iron intake and dietary patterns due to the time and space constraints of the school setting. In future studies, a food frequency questionnaire should be used to yield a more objective evaluation of nutrient intake. Despite these limitations, our findings regarding the prevalence of ID and IDA and their correlates still provide useful local population data, as this is the first study of healthy adolescents in our community.

## 5. Conclusions

The prevalence of ID was found to be 11.1% among adolescent students in Hong Kong. The rates of ID and IDA among local female adolescents were relatively high, at 17.1% and 10.9%, respectively. The individuals with poor dietary habits and girls who reported heavy menstrual bleeding had lower serum ferritin levels. Lower iron stores were associated with worse physical functioning, school functioning, and general fatigue. Even though the prevalence of IDA was relatively low, the potential health consequences of ID without anemia for the physical health and well-being of adolescents should be clearly communicated to the Hong Kong population. Future research should aim to evaluate the effects of iron supplementation on functional outcomes in adolescents with ID or IDA.

## Figures and Tables

**Table 1:** Demographics and dietary characteristics of participants (*n* = 523).

Characteristics	All (*n* = 523) *n* (%)	Male (*n* = 183) *n* (%)	Female (*n* = 340) *n* (%)
**Age** [mean SD]	17.55 [1.05]	17.47 [1.02]	17.59 [1.06]
16–16.9 years	185 (35.4)	69 (37.7)	116 (34.1)
17–17.9 years	182 (34.8)	64 (35.0)	118 (34.7)
18–18.9 years	85 (16.2)	27 (14.7)	58 (17.1)
19–19.9 years	71 (13.6)	23 (12.6)	48 (14.1)
**Weight status**			
Underweight (≤10th percentile)	66 (12.6)	28 (15.3)	38 (11.2)
Normal (10 to <85th percentile)	386 (73.8)	117 (63.9)	269 (79.1)
Overweight (from ≥85th to <95th percentile)	43 (8.2)	17 (9.3)	26 (7.6)
Obese (≥95th percentile)	28 (5.4)	21 (11.5)	7 (2.1)
**Menstrual characteristics (Female)**			
**Age menarche** [mean SD] years	-	-	12.17 [1.42]
**Duration of flow** [mean SD] days	-	-	5.90 [2.96]
**Menstrual cycle** [mean SD] days	-	-	32.00 [14.31]
**Highest amount of sanitary products used on the heaviest day** [mean SD]	-	-	3.98 [1.41]
**Average bleeding per cycle**			
Very little bleeding	-	-	1 (0.3)
Low bleeding	-	-	21 (6.2)
Moderate bleeding	-	-	266 (78.7)
Heavy bleeding	-	-	46 (13.6)
Very heavy bleeding	-	-	4 (1.2)
**Dietary characteristics**			
**Regular habit of skipping ≥ 1 meal**	189 (36.3)	51 (28.4)	138 (40.6)
Skip breakfast	149 (28.5)	45 (24.6)	104 (30.7)
Skip lunch	29 (5.6)	7 (3.8)	22 (6.5)
Skip dinner	41 (7.9)	6 (3.3)	35 (10.3)
**Dietary iron score #** [mean SD]	97.78 [16.10]	99.00 [16.44]	97.13 [15.90]

# The Dietary Iron Score is a composite of the self-reported types and frequency of food intake. Each type of food is ranked based on their general iron content per serving (according to information provided by the Hong Kong Department of Health). A higher score is indicative of higher dietary iron content. More details on the Dietary Iron Score are presented in Supplementary Table S2.

**Table 2:** Rates of iron deficiency and iron deficiency anemia.

	All (*n* = 523) *n* (%)	Male (*n* = 183) *n* (%)	Female (*n* = 340) *n* (%)
Iron deficiency (WHO definition) #	58 (11.1)	0	58 (17.1)
Iron deficiency anemia (WHO definition) ^	37 (7.1)	0	37 (10.9)
Iron deficiency anemia (Local definition) *	23 (4.4)	0	23 (6.8)

WHO: World Health Organization. # Iron deficiency is defined as a serum ferritin level of <15 μg/L for both boys and girls. ^ Iron deficiency anemia is the presence of iron deficiency and anemia, as defined by hemoglobin level of <12 g/dL in girls and a hemoglobin level of <13 g/dL in boys (WHO definition). * ^ Iron deficiency anemia is the presence of iron deficiency and anemia, as defined by hemoglobin level of <11.5 g/dL in girls and a hemoglobin level of <12 g/dL in boys (Local definition).

**Table 3:** Association of serum ferritin with dietary characteristics and menstrual characteristics.

	Descriptive Data	Multivariable Analysis	
Dietary Characteristics	Serum Ferritin Level Median (IQR) μg/L	Estimate (Standard Error)	*p ^#^*
Regular habit of skipping ≥ 1 meal			
Yes (*n* = 189, 36.3%)	54.5 (23.1–105.0)	−35.2 (14.7)	**0.017**
No (*n* = 334, 63.7%)	73.9 (39.2–136.2)	Reference group	
Regular habit of skipping breakfast			
Yes (*n* = 149, 28.5%)	56.1 (28.0–109.0)	−36.8 (15.6)	**0.019**
No (*n* = 374, 71.5%)	73.0 (36.9–133.1)	Reference group	
Regular habit of skipping lunch			
Yes (*n* = 29, 5.5%)	37.8 (16.0–63.2)	−77.3 (30.5)	**0.011**
No (*n* = 495, 94.5%)	71.2 (35.2–129.9)	Reference group	
Regular habit of skipping dinner			
Yes (*n* = 41, 7.8%)	45.8 (17.8–86.8)	−11.4 (26.4)	0.66
No (*n* = 482, 92.2%)	71.2 (35.2–128.2)	Reference group	
Dietary iron score *	-	0.74 (0.43)	0.091
**Weight Status**	**Serum Ferritin Level Median (IQR) μg/L**	**Estimate (Standard Error)**	***p ^#^***
Underweight (*n* = 66, 12.6%)	72.0 (32.0–145.9)	4.9 (21.1)	0.81
Overweight/obese (*n* = 71, 13.6%)	106.8 (40.9–235.8)	82.8 (20.8)	**<0.0001**
Normal weight (*n* = 386, 73.8%)	64.9 (31.1–109.9)	Reference group	
**Menstrual Characteristics (Girls only)**	**Serum Ferritin Level Median (IQR) μg/L**	**Estimate (Standard Error)**	***p*** **^**
Bleeding severity			
Heavy/very heavy bleeding (*n* = 50, 14.7%)	34.0 (20.0–56.0)	−40.8 (20.2)	**0.043**
From very little to moderate bleeding (*n* = 288, 84.7%)	45.8 (20.9–77.4)	Reference group	
Average amount of sanitary products on the heaviest day of the cycle *	-	−13.7 (5.1)	**0.0072**

^#^ Multivariable analysis examining the association between serum ferritin levels and dietary patterns, weight status, adjusted for age and sex. ^ Multivariable analysis examining the association between serum ferritin levels and menstrual characteristics, adjusted for age. * Analyzed as a rank/continuous variable in the multivariable analysis. Boldface indicates statistical significance at *p* < 0.05.

**Table 4:** Factors associated with quality of life and fatigue measures.

	Physical			Emotion			Social			School		
	Est.	SE	*p*	Est.	SE	*p*	Est.	SE	*p*	Est.	SE	*p*
**Serum ferritin** (per 10 μg/L)	0.78	0.27	**0.0047**	0.41	0.42	0.32	0.036	0.32	0.91	0.81	0.40	**0.045**
**Habit of skipping meals**												
Yes	−3.47	1.10	**0.0017**	−3.69	1.65	**0.026**	−1.24	1.33	0.35	−3.85	1.73	**0.027**
No	Ref			Ref			Ref			Ref		
**Dietary iron score**	0.10	0.032	**0.0014**	0.036	0.049	0.46	0.032	0.039	0.41	0.13	0.051	**0.010**

Models are adjusted for age and sex. Est.: estimate; Ref.: reference group; SE: standard error. Boldface indicates statistical significance at *p* < 0.05.

**Table 5:** Factors associated with fatigue measures.

	General Fatigue			Sleep Fatigue			Cognitive Fatigue		
	Est.	SE	*p*	Est.	SE	*p*	Est.	SE	*p*
**Serum ferritin** (per 10 μg/L)	0.92	0.38	**0.016**	0.10	0.42	0.81	−0.14	0.34	0.66
**Habit of skipping meals**									
Yes	−2.58	1.55	0.097	−4.04	1.39	**0.0038**	−4.04	1.39	0.82
No	Ref			Ref			Ref		
**Dietary iron score**	0.06	0.04	0.17	0.02	0.04	0.56	0.09	0.05	0.061

Models are adjusted for age and sex. Est: estimate; Ref: reference group; SE: standard error. Boldface indicates statistical significance at *p* < 0.05.

## Data Availability

The data presented in this study are available on request from the corresponding author. The data are not publicly available due to patient privacy concerns.

## Supplementary Materials

The following supporting information can be downloaded at: https://www.mdpi.com/article/10.3390/ijerph20032578/s1, Supplementary Figure S1: Study Schema; Supplementary Table S1: Instrument Specifications; Supplementary Table S2: Frequency of Intake of Iron-containing Foods; Supplementary Table S3: Summary of Hematological Parameters; Supplementary Table S4: Quality of Life and Fatigue Measures.
