# Supplementary material for: Iron Deficiency among School-Aged Adolescents in Hong Kong: Prevalence, Predictors, and Effects on Health-Related Quality of Life
Source: Int J Environ Res Public Health. 2023 Jan 31;20(3):2578. doi: 10.3390/ijerph20032578 (PMC9916133; doi:10.3390/ijerph20032578)
Supplement: Supplementary file 1 [file ijerph-20-02578-s001.zip › ijerph-2162532-supplementary.pdf]

Supplementary Materials

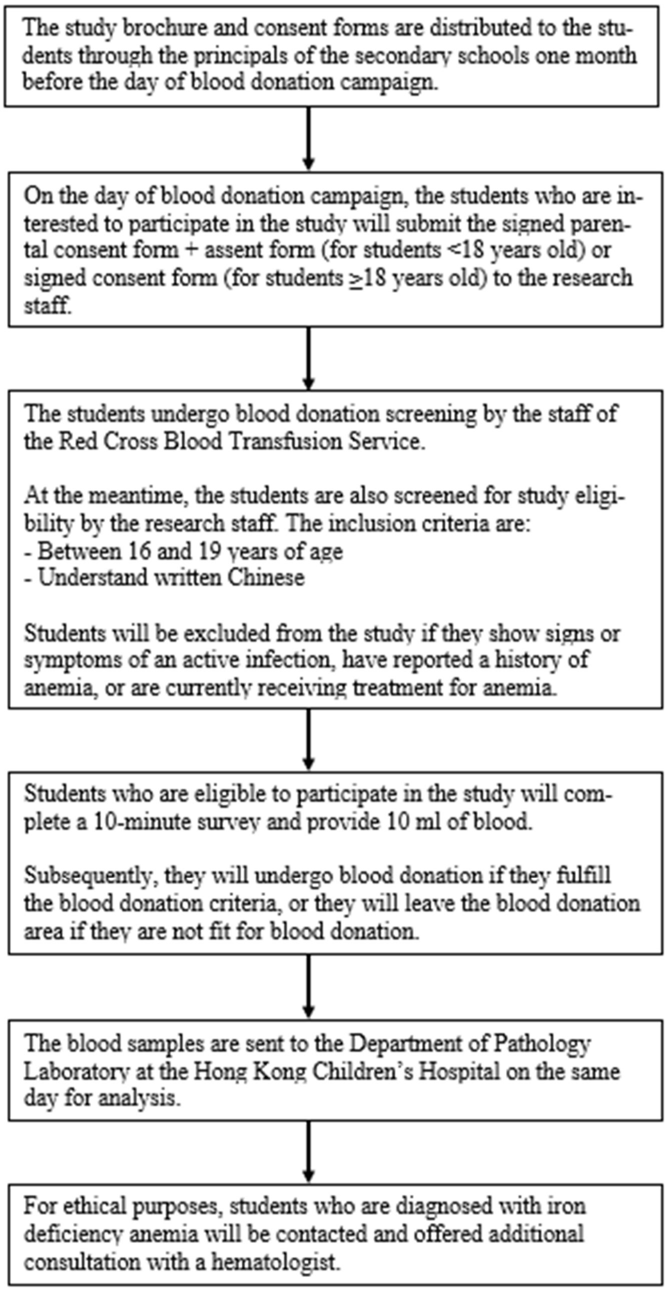

Supplementary Figure S1. Study Schema.

Supplementary Table S1. Instrument Specifications.

**Ferritin**		
Analytical platform: Roche cobas 8000, method: Electrochemiluminescence immunoassay (ECL)		
Within Batch Precision Biorad Tumor Marker QC lot 23960		
Level	Mean	%CV
1	21.9	1.59
2	282	1.03
3	541	1.35
Between Days Precision Biorad Tumor Marker QC lot 94910		
Level	Mean	%CV
1	24.8	2.26
2	302	2.29
3	545	2.70
**C-reactive protein**		
Analytical platform: Roche cobas 8000, method: Particle-enhanced immunoturbidimetric		
Within Batch Precision Biorad Immunology QC lot 68590		
Level	Mean	%CV
1	7.67	1.1
2	24.2	1.5
3	41.4	1.9
Between days Precision Biorad Immunology QC lot 68990		
Level	Mean	%CV
1	8.11	2.41
2	28.7	2.48
3	43.4	2.66
**Iron**		
Analytical platform: Roche cobas 8000, method: Ferrozine, no deproteniniztation		
Within Batch Precision Biorad Multiqual Unassayed QC lot 47990		
Level	Mean	%CV
1	12.8	1.71
2	28.9	2.00
3	42.2	2.00
Between Days Precision Biorad Multiqual Unassayed QC lot 56680		
Level	Mean	%CV
1	13.5	1.84
2	27.1	3.96
3	39.7	1.29
**TIBC = Iron + UIBC**		
UICB:		
Analytical platform: Roche cobas 8000, method: Ferrozine		
Within Batch Precision Biorad Multiqual Unassayed QC lot 47990		
Level	Mean	%CV
1	23.9	3.70
2	21.8	4.58
3	24.5	4.16
Between Days Precision UIBC Biorad Multiqual Unassayed QC lot 56680		
Level	Mean	%CV
1	19.6	6.40
2	35.4	3.18
3	36.1	3.67
**CBC**		

Analyzer XN 9100 L (SIN 14124), XN 9100R (SIN 14122) and XN 1500 (SIN 14121)						
	**QC levels**	**Mean**	**Greatest SD**	**Greatest CV**	**Intra-individual biological variation (CVi)**	**Estimation of uncertainty of measurement (2 CV)**
Hemoglobin (g/dl)	L1	5.7	0.1	1.40		±2.8%
	L2	12	0.1	0.80	2.7%	±1.6%
	L3	15.1	0.1	0.61		±1.22%
WBC (×10⁹/L)	L1	3.02	0.80	2.77		±5.54%
	L2	6.98	0.13	1.86	10.8%	±3.72%
	L3	16.28	0.25	1.54		±3.08%
Platelet (×10⁹/L)	L1	83.4	3	3.69		±7.38%
	L2	263.4	8	3.12	5.6%	±6.24%
	L3	549.2	13	2.44		±4.88%
MCV (fl)	L1	71.6	0.8	1.10		±2.2%
	L2	79.3	0.9	1.10	0.8%	±2.2%
	L3	85.9	0.8	0.90		±1.8%
MCH (pg)	L1	24.3	0.3	1.30		±2.6%
	L2	27.1	0.3	0.90	0.8%	±1.8%
	L3	29.9	0.3	0.90		±1.8%
MCHC (g/dL)	L1	33.9	0.6	1.90		±3.8%
	L2	34.2	0.4	1.20	1.0%	±2.4%
	L3	34.8	0.5	1.30		±2.6%
HCT (L/L)	L1	0.168	0.003	1.83		±3.66%
	L2	0.349	0.004	1.22	2.8%	±2.44%
	L3	0.434	0.006	1.22		±2.44%
RDW (%)	L1	18.5	0.3	1.50		±3.0%
	L2	17.9	0.2	1.10	4.3%	±2.2%
	L3	16.3	0.11	0.70		±1.4%
Reticulocyte (×10⁹/L)	L1	138.08	5.00	3.73		±7.46%
	L2	108.8	4.74	4.49	9.7%	±8.98%
	L3	65.362	3.54	5.60		±11.2%

Courtesy of the Department of Pathology, Hong Kong Children's Hospital.

Supplementary Table S2. Frequency of Intake of Iron-containing Foods.

**Dietary Characteristics**	**Estimated Iron Content #**	**All (n = 523)** *n* **(%)**	**Male (n = 183)** *n* **(%)**	**Female (n = 340)** *n* **(%)**
**Intake of seafood** [8]				
Not at all		47 (9.0)	15 (8.2)	32 (9.4)
Once/twice per week	9.2 – 27.9 mg (100mg)	272 (52.1)	89 (48.6)	183 (54.0)
Alternate day		150 (28.7)	62 (33.9)	88 (26.0)
Daily		53 (10.2)	17 (9.3)	36 (10.6)
**Intake of meat** [7]				
Not at all		1 (0.2)	1 (0.6)	0
Once/twice per week	1.2 – 2.6 mg (100g)	26 (5.0)	3 (1.6)	23 (6.8)
Alternate day		92 (17.6)	25 (13.7)	67 (19.8)
Daily		403 (77.2)	154 (84.2)	249 (73.4)
**Intake of iron-fortified cereal** [6]				
Not at all		106 (20.3)	42 (23.0)	64 (18.9)
Once/twice per week	1.8 mg (40 g)	134 (25.7)	40 (21.9)	94 (27.7)
Alternate day		68 (13.0)	14 (7.7)	54 (15.9)
Daily		214 (41.0)	87 (47.5)	127 (37.5)

**Intake of leafy vegetables (e.g. spinach)** [5]				
Not at all		18 (3.5)	8 (4.4)	10 (3.0)
Once/twice per week	6.4 g (180 g)	88 (16.9)	36 (19.7)	52 (15.3)
Alternate day		109 (20.9)	38 (20.8)	71 (20.9)
Daily		307 (58.8)	101 (55.2)	206 (60.8)
**Intake of beans** [4]				
Not at all		104 (19.9)	29 (15.9)	75 (22.1)
Once/twice per week	4.6 – 8.8 mg (1 cup)	303 (58.1)	112 (61.2)	191 (56.3)
Alternate day		93 (17.8)	32 (17.5)	61 (18.0)
Daily		22 (4.2)	10 (5.5)	12 (3.5)
**Intake of nuts** [3]				
Not at all		200 (38.5)	70 (38.3)	130 (38.6)
Once/twice per week	1.2 – 1.7 mg (1 oz)	259 (49.8)	91 (49.7)	168 (49.9)
Alternate day		37 (7.1)	15 (8.2)	22 (6.5)
Daily		24 (4.6)	7 (3.8)	17 (5.0)
**Intake of dry fruits** [2]				
Not at all		329 (63.0)	109 (59.6)	220 (64.9)
Once/twice per week	1.3 – 1.7 mg (0.5 cup)	168 (32.2)	68 (37.2)	100 (29.5)
Alternate day		19 (3.6)	5 (2.7)	14 (4.1)
Daily		6 (1.2)	1 (0.6)	5 (1.5)
**Intake of eggs** [1]				
Not at all		9 (1.7)	2 (1.1)	7 (2.1)
Once/twice per week	0.9 mg (1 piece)	150 (28.8)	47 (25.8)	103 (30.4)
Alternate day		234 (44.9)	82 (45.1)	152 (44.8)
Daily		128 (24.6)	51 (28.0)	77 (22.7)
**Dietary iron score*** [mean SD]		97.78 [16.10]	99.00 [16.44]	97.13 [15.90]

Estimated iron content in common foods taken by the Hong Kong population. This information is provided by the Department of Health, The Government of the Hong Kong Special Administrative Region. * The dietary iron score is a composite of the type and frequency of food intake. Each type of food is ranked based on the estimated iron content per serving (shown in the Table). The rankings are presented as numerical subscripts (1 to 8), with a higher number indicative of higher iron content. For example, seafood has the highest iron content per serving (8 points) while egg has the lowest iron content per serving (1 point). The frequency of intake is graded from 1 to 4 points (1 point=Not at all, 2 points=Once/twice per week; 3 points=Alternate day; 4 points=Daily). The dietary iron score is tabulated by the formula: *(ranking of food 1)*(the frequency of intake of food 1) + (ranking of food 2)*(the frequency of intake of food 2) + ….* For example, if an adolescent reported taking seafood once/twice per week and beans on alternate days, he will be scored as (8*3) + (5*3) = 39 points. The total computed score ranges from 36 points to 144 points.

Supplementary Table S3. Summary of Hematological Parameters.

	Mean	SD	Median	IQR
**All**				
Hemoglobin (g/dl)	13.3	1.4	13.1	12.3 - 14.4
WBC (×10^9/L)	6.9	1.5	6.9	5.9 - 7.9
Platelet (x10^9/L)	259.2	59.5	255.0	216.0 - 293.5
MCV (fl)	86.2	6.6	87.4	84.7 - 89.8
MCH (pg)	28.5	4.4	28.9	27.9 - 29.8
MCHC (g/dL)	32.7	1.1	32.8	32.2 - 33.5
HCT (L/L)	0.4	0.03	0.40	0.37 - 0.43
RDW (%)	12.7	1.4	12.3	11.9 - 12.9
Reticulocyte (%)	1.5	0.4	1.4	1.2 - 1.7
Ferritin (µg/L)	94.6	86.7	69.9	33.0 - 124.2
**Male Adolescents**				
Hemoglobin (g/dl)	14.7	0.8	14.7	14.2 - 15.2
WBC (×10^9/L)	6.7	1.5	6.6	5.6 - 7.5
Platelet (x10^9/L)	246.5	52.3	244	212 - 274
MCV (fl)	85.7	6.3	87.0	84.8 - 88.9
MCH (pg)	29.0	6.2	29.0	28.3 - 29.8
MCHC (g/dL)	33.1	0.9	33.3	32.6 - 33.8
HCT (L/L)	0.44	0.02	0.44	0.43 - 0.45
RDW (%)	12.5	1.4	12.2	11.8 - 12.6
Reticulocyte (%)	1.5	0.4	1.4	1.1 - 1.7
Ferritin (µg/L)	160.9	92.1	136.1	89.8 - 219.8
**Female Adolescents**				
Hemoglobin (g/dl)	12.5	0.9	12.5	11.9 - 13.1
WBC (×10^9/L)	7.0	1.4	7.0	6.0 - 7.9
Platelet (×10^9/L)	266.0	62.0	266	219 - 305
MCV (fl)	86.4	6.8	88.1	84.6 - 90.3
MCH (pg)	28.1	2.9	28.8	27.4 - 29.8
MCHC (g/dL)	32.5	1.0	32.6	32.1 - 33.2
HCT (L/L)	0.38	0.02	0.38	0.36 - 0.40
RDW (%)	12.7	1.3	12.4	12.0 - 13.1
Reticulocyte (%)	1.4	0.4	1.4	1.1 - 1.7
Ferritin (µg/L)	58.9	58.1	44.9	20.9 - 74.5

HCT: hematocrit; MCH: mean corpuscular hemoglobin; MCHC: mean corpuscular hemoglobin concentration; MCV: mean corpuscular volume; RDW: red cell distribution width; WBC: white blood cell.

Supplementary Table S4. Quality of Life and Fatigue Measures.

**Quality of Life**	**All (n = 523)**
**Physical** *	84.77 (12.33)
Emotion	67.78 (18.10)
Social	82.92 (14.47)
School	66.17 (18.91)
**Psychosocial** *	74.92 (13.03)
**Total** *	77.65 (11.95)
**Fatigue**	**All (n = 522) * 1 missing data**
General	73.45 (17.09)
Sleep	61.45 (15.16)
Cognitive	69.11 (18.87)
**Total** #	68.01 (13.87)

A higher score is indicative of better functioning (ie. better quality of life and less fatigue). * The "Psychosocial subscale" consists of the Emotion, Social and School subdomains. The "Total scale" consists of the Physical, Emotion, Social, and School subdomains. # The "Total fatigue scale" consists of the General, Sleep and Cognitive fatigue subdomains.
